# Lack of Association Between Middle Cerebral Artery Diastolic Deceleration Area and Gestational Diabetes Mellitus: A Prospective Case-Control Study

**DOI:** 10.3390/medicina62050957

**Published:** 2026-05-14

**Authors:** Zubeyde Emiralioglu Cakır, Hale Ankara Aktaş, Ilayda Gercik Arzık, Ceren Saglam, İlker Cakir, İlknur Toka, Mükremin Ceylan, Pınar Tuğçe Özer, Hakan Golbasi

**Affiliations:** 1 Division of Perinatology, Department of Obstetrics and Gynecology, Buca Seyfi Demirsoy Education and Research Hospital, İzmir 35390, Turkey; 2 Department of Perinatology, Izmir City Hospital, Izmir 35540, Turkey; haleankara@gmail.com (H.A.A.); mukreminceylan68@gmail.com (M.C.);; 3 Division of Gynecologic Oncology, Department of Obstetrics and Gynecology, Buca Seyfi Demirsoy Education and Research Hospital, İzmir Democracy University Faculty of Medicine, İzmir 35390, Turkey; 4 Department of Obstetrics and Gynecology, İzmir Ekonomi Üniversitesi Medical Point Hospital, İzmir 35575, Turkey; pintugbar@gmail.com

**Keywords:** gestational diabetes mellitus, fetal doppler ultrasound, MCA DDA, dicrotic notch, ductus venosus, TAMXV, NICU

## Abstract

*Backgroud and Objectives*: To evaluate conventional Doppler indices and the novel middle cerebral artery (MCA) diastolic deceleration area (DDA) in pregnancies complicated by gestational diabetes mellitus (GDM), and to explore their associations with perinatal outcomes. Prospective case–control study conducted at a tertiary referral perinatology center. *Materials and Methods*: The study included 83 women with GDM and 92 healthy controls. Standard fetal biometric and Doppler parameters—umbilical artery, MCA, ductus venosus, cerebroplacental ratio, and umbilicocerebral ratio—were assessed, alongside calculation of MCA DDA. Perinatal outcomes were recorded. *Results*: Most conventional Doppler indices did not differ between groups, except for lower MCA dicrotic notch velocity and higher ductus venosus time-averaged maximum velocity in the GDM group. MCA DDA values did not differ significantly between GDM and control groups (6.67 [5.02–8.20] vs. 7.05 [5.21–8.39] cm·s, *p* = 0.444) and showed no difference between insulin- and diet-controlled subgroups (*p* > 0.05). MCA DDA showed significant correlations with gestational age, MCA peak systolic velocity, and birth weight. However, after adjustment for potential confounders, gestational age remained the only independent determinant of MCA DDA. The multivariable analysis evaluating composite adverse neonatal outcomes was limited by the small number of adverse events (*n* = 14). *Conclusions*: MCA DDA did not differ between GDM and control pregnancies and primarily reflected gestational age-related physiological variation rather than diabetes specific hemodynamic changes. However, its relationship with adverse neonatal outcomes remains uncertain and requires further investigation in larger prospective studies.

## 1. Introduction

Gestational diabetes mellitus (GDM) is defined as glucose intolerance that first develops or is first recognized during pregnancy. It is associated with increased maternal and fetal risks throughout gestation. Prenatal complications linked to GDM include polyhydramnios, macrosomia, neonatal hypoglycemia, hyperbilirubinemia, hypocalcemia, and hypomagnesemia [1]. Offspring of GDM pregnancies are also at increased risk of developing obesity, metabolic syndrome, hypertension, and diabetes later in life [2].

Doppler ultrasonography has been extensively investigated to assess fetal well-being in diabetic pregnancies. These include commonly used indices, such as umbilical artery (UA), middle cerebral artery (MCA), and ductus venosus (DV) Doppler indices, as well as the cerebroplacental ratio (CPR) and umbilicocerebral ratio (UCR). Additional measures, such as myocardial performance index and hepatic or pulmonary artery Dopplers, have also been evaluated [2,3,4,5,6,7,8,9].

More recently, a novel parameter, the MCA diastolic deceleration area (DDA), has been introduced as a predictor of neonatal outcome. MCA DDA reflects end-systolic pressure and peripheral vascular resistance, offering insights into cerebral vasodilation and the brain-sparing effect. It has been shown to correlate strongly with umbilical cord pH and, particularly when pH ≤ 7.21, to predict hypoxemia with high sensitivity and specificity. Accordingly, MCA DDA has been proposed as an independent marker of fetal hypoxia [10].

Given the distinct hemodynamic and metabolic alterations in GDM, studying MCA DDA in this context may provide novel insights. The present study aimed to evaluate both conventional Doppler parameters and MCA DDA in pregnancies complicated by GDM compared with healthy controls. We further assessed the associations between these indices and perinatal outcomes to determine whether MCA DDA provides additional insights into fetal adaptation and neonatal morbidity in this population or whether it shows no clinically meaningful association with GDM.

## 2. Materials and Methods

This prospective, case-control study was conducted at the Perinatology Clinic of Izmir City Hospital between 10 March 2025 and 10 August 2025. Approval was obtained from the local ethics committee (Approval code: 2025/123, 7 March 2025), and the study was carried out in accordance with the Declaration of Helsinki. Written informed consent was obtained from all participants. Participants were recruited consecutively during the study period from the perinatology clinic, and all eligible patients were included according to predefined inclusion criteria, minimizing potential selection bias.

All participants underwent a 75-g oral glucose tolerance test (OGTT) between 24 and 28 weeks of gestation. According to the International Association of Diabetes and Pregnancy Study Groups criteria, GDM was diagnosed when fasting plasma glucose was ≥92 mg/dL (5.1 mmol/L), 1-h glucose ≥ 180 mg/dL (10.0 mmol/L), or 2-h glucose ≥ 153 mg/dL (8.5 mmol/L) [11]. Women who did not meet these thresholds were included in the control group. GDM cases were further classified as A1 (diet-controlled) or A2 (insulin-requiring) [].

Eligible participants were women with singleton pregnancies between 28 and 40 weeks of gestation. Exclusion criteria were multiple pregnancies, congenital anomalies, pregestational diabetes, other metabolic disorders, gestational hypertension or preeclampsia, medication use, and fetal growth restriction(FGR). Maternal age, gestational weight gain, body mass index (BMI), and reproductive history (gravidity, parity, abortion) were recorded. GA was confirmed in all participants by first-trimester crown–rump length (CRL) measurement.

Sample size calculation was performed using G*Power 3.1.9.2. Based on an effect size of 0.440 from a previous study [12] with α = 0.05 and 80% power, a minimum of 82 participants per group (total *n* = 164) was required. Additionally, a sensitivity power analysis using the actual sample size demonstrated 82.4% power to detect an effect size of d = 0.44.

All ultrasound examinations were performed with women in the supine position using a Voluson E8 system (GE Healthcare, Wauwatosa, WI, USA) equipped with a 2–9 MHz convex transducer. All examinations were performed by a single experienced perinatologist (Z.E.C.) to minimize interobserver variability. To assess intraobserver reproducibility of MCA DDA measurements, repeated measurements were performed in 20 randomly selected cases by the same observer at two different time points separated by a 30-min interval. Intraobserver reproducibility was evaluated using the intraclass correlation coefficient (ICC), calculated with a two-way mixed-effects model based on absolute agreement. ICC values were interpreted as follows: <0.50 poor, 0.50–0.75 moderate, 0.75–0.90 good, and >0.90 excellent reliability. Intraobserver reproducibility analysis demonstrated excellent agreement for MCA DDA measurements, with an ICC of 0.91 (95% CI: 0.80–0.96; *p* < 0.001).

All biometric parameters, including biparietal diameter (BPD), head circumference (HC), abdominal circumference (AC), and femur length (FL), were measured in accordance with the International Society of Ultrasound in Obstetrics and Gynecology (ISUOG) Practice Guidelines for ultrasound assessment of fetal biometry and growth [13]. Estimated fetal weight (EFW) was automatically calculated by the ultrasound system according to the Hadlock formula. All fetal biometric measurements, along with their calculated percentiles, were documented according to the Hadlock reference standards [14].

Doppler examinations were performed in accordance with the ISUOG recommendations for the clinical application of Doppler in obstetrics [13]. The umbilical artery (UA), MCA, and ductus venosus (DV) were systematically assessed. For the UA and MCA, both systolic/diastolic ratio (S/D) and pulsatility index (PI) were calculated, with peak systolic velocity (PSV) additionally recorded for the MCA. For the DV, pulsatility index (PI) and time-averaged maximum velocity (TAMXV) were obtained. UA Doppler recordings were taken from a free-floating loop of the cord, avoiding contact with the fetus or placenta. MCA measurements were acquired in an axial view of the fetal head, at the vessel’s origin from the circle of Willis, approximately 2 mm distal to its branching point, maintaining an insonation angle below 30° without exerting pressure on the fetal head. DV waveforms were obtained at the point immediately before the umbilical vein entered the inferior vena cava. The cerebroplacental ratio (CPR) and umbilicocerebral ratio (UCR) were calculated as MCA-PI/UA-PI and UA-PI/MCA-PI, respectively.

All fetal biometric parameters (BPD, HC, AC, FL) were measured in millimeters (mm), and estimated fetal weight (EFW) in grams (g). Doppler velocities were expressed in cm/s.

**Figure 1:**
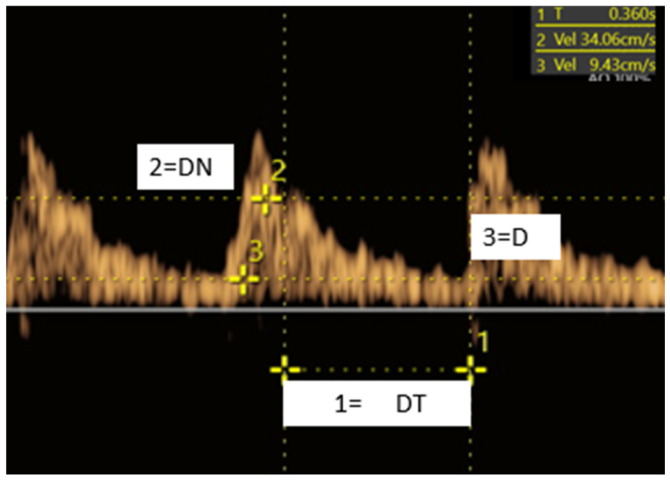
Sonographic assessment method of the middle cerebral artery (MCA) diastolic deceleration area (DDA). 1 = DT, deceleration time (corresponding to T on the device display); 2 = DN, dicrotic notch (corresponding to Vel2 on the device display); 3 = D, end-diastolic velocity (corresponding to Vel3 on the device display).

In the MCA Doppler waveform, the dicrotic notch (DN), which appears on the descending limb and marks the end of systole and the beginning of diastole, and end-diastolic velocity (D), both expressed in m/s, were identified, along with the interval between these two points (Δt = t2 − t1), measured as deceleration time (DT, s). The diastolic deceleration area (DDA) was subsequently calculated using the formula: ½ × (DN + D) × Δt (Figure 1) [10].

Neonatal data, including date of birth (gestational week), birth weight (g), Apgar scores at 1 and 5 min, NICU admission and duration of stay (days), hyperbilirubinemia requiring phototherapy, hypocalcemia, neonatal hypoglycemia, and other morbidities (such as neonatal sepsis and perinatal asphyxia), were recorded. Composite adverse neonatal outcome (CANO) was defined as the presence of one or more of the following: 5th minute Apgar score <7, respiratory distress syndrome (RDS), need for continuous positive airway pressure (CPAP) or mechanical ventilation, neonatal sepsis, perinatal asphyxia, or neonatal intensive care unit (NICU) admission. Neonatal respiratory morbidity was diagnosed based on characteristic clinical signs (tachypnea, chest retractions, nasal flaring, grunting, or cyanosis) in combination with typical radiographic features, such as transient tachypnea of the newborn or a reticulogranular pattern consistent with respiratory distress syndrome (RDS).

All analyses were performed using SPSS software, version 22.0 (SPSS Inc., Chicago, IL, USA). Descriptive data were expressed as numbers and percentages for categorical variables, and as Median (IQR) for continuous variables. Categorical variables were compared between groups using the Pearson chi-square test. The Kolmogorov–Smirnov test was applied to assess the normality of distribution for continuous variables. For comparisons between two independent groups, the Mann–Whitney U test was used. Associations between continuous variables were evaluated with Spearman’s correlation analysis. A *p* value < 0.05 was considered statistically significant. To assess the independent association of MCA DDA with CANO, multiple logistic regression analysis was performed, including MCA DDA, GDM status, maternal age, BMI, gestational age at examination, and insulin use as covariates. Additionally, multiple linear regression analysis was conducted to identify independent predictors of MCA DDA values, with GDM status, maternal age, BMI, gestational age at examination, and insulin use included as independent variables. Results are presented as odds ratios (OR) with 95% confidence intervals for logistic regression, and as standardized coefficients (Beta) with 95% confidence intervals for linear regression.

There were no missing data for maternal, Doppler, or neonatal variables; all enrolled participants were included in the final analyses.

## 3. Results

A total of 175 participants were included in the study, comprising 83 patients and 92 controls. Compared with controls, the patient group had significantly higher maternal age (*p* < 0.001), gravida (*p* = 0.020), number of abortions (*p* = 0.034), weight (*p* < 0.001), and body mass index (BMI) (*p* < 0.001), while GA at delivery was significantly lower (*p* < 0.001). Maternal characteristics and perinatal outcomes of the study and control groups are presented in Table 1.

In the study group, BPD percentile (*p* = 0.009), HC percentile (*p* < 0.001), AC (*p* = 0.011), AC percentile (*p* < 0.001), EFW percentile (*p* < 0.001), and DV TAMXV (*p* = 0.030) were significantly higher than in the control group, whereas DN (*p* = 0.048) was significantly lower. MCA DDA values did not differ significantly between GDM and control groups (6.67 [5.02–8.20] vs. 7.05 [5.21–8.39] cm·s, *p* = 0.444). Table 2 summarizes the ultrasonographic measurements and Doppler indices in both groups.

The rate of cesarean section(CS) was significantly higher in the study group (79.5%) compared with the control group (65.2%) (*p* = 0.035). No significant differences were observed between the groups regarding neonatal outcomes. Table 3 shows the delivery mode and neonatal outcomes in diabetic and control groups.

Doppler parameters were analyzed in relation to insulin treatment status. In the patient group, most Doppler parameters were comparable between insulin-treated and non-insulin-treated women (MCA DDA, UA S/D ratio, PI UA, PSV MCA, PI MCA, S/D MCA, CPR, UCR, and PI DV; for all *p* > 0.05). The only significant difference was observed in DV TAMXV, which was lower in the insulin-treated group compared with those not receiving insulin [38.90 (33.30–50.17) vs. 51.60 (39.70–62.88), *p* = 0.038]. However, when insulin-treated patients were compared with healthy controls, no significant difference in DV TAMXV values was detected (*p* = 0.393).

In the GDM group, MCA DDA showed a weak-to-moderate positive correlation with GA (r = 0.391, *p* < 0.001). Weak positive correlations were found with BPD (r = 0.246, *p* = 0.025), FL (r = 0.316, *p* = 0.004) and EFW(r = 0.236, *p* = 0.032). A moderate positive correlation was observed with PSV MCA (r = 0.570, *p* < 0.001). In contrast, MCA DDA showed weak negative correlations with PI MCA (r = –0.274, *p* = 0.013) and birth weight (r = −0.260, *p* = 0.018). Notably, MCA DDA showed a positive correlation with NICU stay duration in newborns of mothers with GDM (r = 0.880, *p* = 0.021). Table 4 shows the correlations between MCA DDA and other clinical and Doppler parameters in the GDM group.

In the GDM group, MCA DDA values did not differ significantly by mode of delivery (CS vs. NVD), presence of neonatal respiratory morbidity, or CANO (all *p* > 0.05, Mann–Whitney U test) (Table 4). A significant correlation was observed between MCA DDA and NICU stay duration (r = 0.880, *p* = 0.021); however, this correlation was calculated exclusively among the seven neonates admitted to the NICU in the GDM group.

In the GDM group, GA at delivery showed a weak negative correlation with PI DV (r = −0.239, *p* = 0.030). Birth weight demonstrated a weak negative correlation with MCA DT (r = −0.237, *p* = 0.031) and MCA DDA (r = −0.260, *p* = 0.018), whereas weak positive correlations were observed with PI MCA (r = 0.235, *p* = 0.034), S/D MCA (r = 0.262, *p* = 0.017), and CPR (r = 0.244, *p* = 0.027). A weak negative correlation was also found between birth weight and UCR (r = −0.244, *p* = 0.027). The 5-min Apgar score showed a weak negative correlation with MCA DN (r = −0.248, *p* = 0.024). These correlations are summarized in Table 5.

To identify the independent factors affecting MCA DDA, multiple linear regression analysis was performed. The model explained 13.2% of the total variance in MCA DDA values (R^2^ = 0.132; adjusted R^2^ = 0.107; F(5,169) = 5.151; *p* < 0.001). VIF values ranged from 1.01 to 1.91, indicating no multicollinearity. After adjustment for confounders, gestational age was the only independent determinant of MCA DDA (β = 0.343; *p* < 0.001). GDM status (*p* = 0.469), maternal age (*p* = 0.601), BMI (*p* = 0.109), and insulin use (*p* = 0.564) showed no independent association (Table 6).

Multiple logistic regression analysis was performed to evaluate the independent effect of MCA DDA on CANO, including MCA DDA, GDM status, maternal age, BMI, gestational age, and insulin use as covariates. The overall model was not statistically significant (χ^2^ = 1.802; df = 6; *p* = 0.937; Nagelkerke R^2^ = 0.024). No variable was identified as an independent predictor, including MCA DDA (OR = 0.953; 95% CI: 0.753–1.205; *p* = 0.687). This finding should be interpreted with caution due to the low number of events (n = 14), which may limit its robustness (Table 7).

## 4. Discussion

In this study, we found no significant difference in the novel MCA DDA between pregnancies complicated by GDM and healthy controls. Most conventional Doppler indices showed no significant differences between groups, except for MCA DN and DV TAMXV. Birth weight was positively correlated with CPR, MCA PI, and MCA S/D, but negatively correlated with UCR, MCA DT, and MCA DDA. Furthermore, MCA DN showed a negative correlation with the 5-min Apgar score. Importantly, multivariable linear regression analysis revealed that GA was the only independent determinant of MCA DDA, while GDM status had no significant effect.

In line with our findings, conventional Doppler indices showed limited predictive value in GDM. Previous studies have reported conflicting results: some demonstrated weak associations of CPR or CPUR with adverse outcomes [5,6,15], while others described lower MCA PSV or side-specific MCA PI differences in GDM fetuses [16,17]. Conversely, several studies found no significant predictive value for UA-PI, MCA-PI, or MCA-PSV [4,18]. Acar et al. reported no differences in most Doppler indices except for hepatic artery parameters, although correlations with HbA1c suggested that poor glycemic control may contribute to chronic hypoxia [2]. In our cohort, no significant differences were observed between the GDM and control groups regarding UA or MCA Doppler indices, CPR, or UCR, and no associations were found with neonatal outcomes; however, weak, parameter-specific correlations with birth weight were observed.

Apart from arterial Dopplers, venous indices such as the DV have also been investigated as indicators of fetal cardiac function. Increased DV-PI and abnormal atrial contraction flow have been linked to adverse outcomes, though findings in diabetic pregnancies are inconsistent in diabetic pregnancies [2,19]. DV diameter is often enlarged, whereas DV TAMXV remains unchanged or reduced [3,20]. Consequently, normalized DV flow decreases, suggesting reduced DV shunting and increased hepatic perfusion, possibly reflecting suppression of normal adaptive mechanisms by fetal hyperglycemia [3]. In our study, DV-PI decreased with GA as expected. Interestingly, DV TAMXV was significantly higher in GDM compared to controls, but lower in insulin-treated cases than in diet-controlled ones, with values similar to non-diabetic pregnancies. These results suggest that while adaptations are largely preserved in GDM, they may be impaired in insulin-requiring cases, reflecting the impact of poorer glycemic control. These findings may suggest subtle differences in venous adaptation according to glycemic control; however, given the exploratory nature of the analyses and the absence of correction for multiple comparisons, the findings should be interpreted cautiously. Further studies with larger cohorts and dedicated validation analyses are needed to confirm these observations.

Because conventional Dopplers have limited predictive power, novel indices such as MCA DDA have been proposed [10]. To date, this parameter has primarily been investigated in late-onset FGR among high-risk pregnancies. In a study including 45 FGR cases and 45 controls, MCA DDA values were significantly higher in the FGR group. In the same study, CPR and UCR exhibited high sensitivity but low specificity for adverse outcomes, while CPUR showed more balanced performance, and DDA emerged as the parameter with the highest specificity [21]. More recently, a cohort study including 51 FGR cases and matched controls reported a moderate ability of MCA DDA to predict adverse perinatal outcomes, comparable to that of conventional Doppler indices such as CPR and CPUR, and suggested that integrating MCA DDA with established Doppler parameters may improve fetal surveillance in pregnancies complicated by FGR [12]. Additionally, in studies evaluating quantitative ultrasound-derived obstetric parameters, methodological validation with standardized acquisition protocols and reproducibility analyses has been considered essential before wider clinical application [22]. However, although previous studies have confirmed good intraobserver reproducibility of MCA DDA measurements (ICC = 0.911) [10], formal interobserver variability assessment remains limited. Therefore, further studies, including comprehensive reproducibility analyses, are still needed before broader clinical implementation of MCA DDA.

To our knowledge, this is the first study to evaluate MCA DDA in pregnancies complicated by GDM. In contrast to its reported performance in FGR [12,21], MCA DDA did not differ between GDM and control pregnancies, nor between insulin- and diet-treated cases. However, MCA DDA showed positive correlations with GA, BPD, FL, EFW, and a negative correlation with MCA PI. These findings suggest that MCA DDA primarily reflects GA-dependent physiological changes, which is in line with previous reports indicating that MCA DDA increases with advancing gestation. The absence of associations with fetal biometric percentiles and the very weak correlation with EFW, together with the lack of correlation with AC, further support the notion that MCA DDA is not a marker of macrosomia. In addition, MCA DDA showed a negative correlation with birth weight, whereas MCA PI and S/D ratios correlated positively. These findings likely reflect subtle hemodynamic adaptations rather than determinants of neonatal size. Larger, multicenter studies are needed to clarify their clinical significance.

Our study also analyzed the individual parameters contributing to the calculation of MCA DDA. Among these, the DN velocity was significantly lower in the GDM group compared to controls. Although the exact mechanism of DN formation is unclear [10,23]. It has been proposed that the DN results from the reflection of a backward wavefront at aortic valve closure. More recent studies have proposed alternative mechanisms: one attributed it to transient arterial wall pressure fluctuations caused by multidirectional acceleration of the aortic valve apparatus at closure, whereas another linked it to changes in peripheral vascular resistance [23,24]. The observed decrease in DN velocity may reflect increased vascular stiffness and reduced arterial compliance, which have been described in GDM, and could represent an early marker of maternal diabetes-related metabolic and hemodynamic effects on the fetal circulation. Therefore, these findings may indicate subtle hemodynamic alterations; however, further validation studies are required.

In our study, MCA D showed a weak negative correlation with the 5-min Apgar score, warranting further investigation. Additionally, MCA DDA demonstrated a positive correlation with the duration of NICU stay in newborns born to mothers with GDM. However, after adjustment for potential confounders, GA remained the only independent determinant of MCA DDA. Given the limited number of adverse neonatal outcome events, analyses evaluating the relationship between MCA DDA and neonatal outcomes were statistically constrained. Thus, the observed association between higher MCA DDA values and prolonged NICU stay in newborns born to mothers with GDM should be interpreted with caution, especially considering the limited number of neonates who required NICU admission. Larger prospective studies are required to better elucidate the clinical significance and predictive utility of MCA DDA.

Our study has some limitations. Because this is a single-center study restricted to gestational diabetes pregnancies, the findings may not be fully generalizable to broader populations. Although gestational age comparability was ensured between groups, the lack of full matching for all potential confounding variables may have introduced residual confounding. The lack of hemoglobin A1c measurements limited the ability to objectively assess maternal glycemic control. In addition, cord blood gas analysis was not routinely performed in our institution and was only obtained in cases with suspected intrapartum fetal compromise. This limitation restricts our ability to assess the relationship between MCA DDA and fetal acidemia or hypoxia. Furthermore, the relatively low number of CANO events may limit the statistical power of the analyses and should be considered when interpreting the results. Another limitation is the small number of NICU admissions, which calls for cautious interpretation of NICU-related findings. Although a preliminary intraobserver reproducibility analysis was performed in a subset of 20 randomly selected cases, the relatively small sample size for intraobserver assessment remains a methodological limitation. Additionally, although multiple Doppler parameters were compared in this study, no correction for multiple comparisons was applied, which may increase the risk of Type I error, particularly for findings with borderline significance. Therefore, findings with borderline statistical significance should be considered exploratory and hypothesis-generating. Furthermore, the relatively wide gestational age range may have introduced variability in Doppler measurements and represents a limitation of the study. Future studies with a narrower gestational age window or stratified analyses are needed to better account for gestational age-related physiological variation. Finally, longitudinal changes in Doppler parameters across gestation were not systematically addressed, which may have provided further insights into temporal hemodynamic adaptations.

## 5. Conclusions

This study demonstrates that most conventional Doppler indices, including the novel MCA DDA, do not differ between pregnancies complicated by GDM and healthy controls. MCA DDA primarily reflects gestational age-related physiological changes rather than GDM-specific hemodynamic adaptations. Although MCA DDA showed a positive correlation with NICU stay duration in the GDM group, its relationship with adverse neonatal outcomes remains uncertain and should be interpreted with caution due to the small number of events. Additionally, lower MCA DN velocity in the GDM group and reduced DV TAMXV in insulin-treated cases suggest possible subtle fetal hemodynamic adaptations related to glycemic control. These exploratory findings were not subjected to multivariable adjustment and were not corrected for multiple comparisons; thus, they should be interpreted cautiously and confirmed in larger multicenter studies.

## Figures and Tables

**Table 1:** Maternal characteristics and perinatal outcomes in study and control groups.

	GDM (*n* = 83)	Control (*n* = 92)	*p* *
	Median (IQR)	Median (IQR)	
Maternal age	31.00 (28.00–35.00)	27.00 (25.00–31.00)	**<0.001**
GA	33.00 (32.00–36.00)	34.00 (32.00–36.00)	0.620
Gravida	2.00 (1.00–3.00)	2.00 (1.00–2.00)	**0.020**
Parity	1.00 (0.00–2.00)	1.00 (0.00–1.00)	0.083
Abortions	0.00 (0.00–1.00)	0.00 (0.00–0.00)	**0.034**
Weight (kg)	84.00 (77.00–97.00)	76.00 (66.00–85.50)	**<0.001**
Height (cm)	161.00 (158.00–167.00)	161.00 (158.50–165.50)	0.877
BMI (kg/m^2^)	31.86 (29.76–35.93)	29.12 (25.12–31.98)	**<0.001**
GA at delivery	37.00 (37.00–38.00)	38.00 (38.00–39.00)	**<0.001**
Birth weight	3370.00 (2930.00–3640.00)	3200.00 (2910.00–3375.00)	0.081
APGAR Score at 1st minute	8.00 (8.00–8.00)	8.00 (8.00–8.00)	0.253
APGAR Score at 5th minute	9.00 (9.00–9.00)	9.00 (9.00–9.00)	0.158
NICU stay duration	3.50 (3.00–11.00)	6.00 (5.00–8.00)	0.428

* The Mann–Whitney U test was performed. BMI: body mass index, GA: Gestational age, NICU: neonatal intensive care unit.

**Table 2:** Ultrasonographic measurements and Doppler indices in GDM and control groups.

	GDM (*n* = 83)	Control (*n* = 92)	*p* *
	Median (IQR)	Median (IQR)	
BPD	85.76 (80.25–91.20)	85.70 (79.16–89.69)	0.336
BPD percentile	69.00 (36.10–93.70)	58.40 (30.45–76.20)	**0.009**
HC	315.28 (293.40–331.00)	306.52 (288.14–320.58)	0.053
HC percentile	49.10 (16.40–81.00)	18.70 (9.30–49.20)	**<0.001**
AC	313.53 (286.59–331.20)	301.43 (278.36–319.00)	**0.011**
AC percentile	84.30 (41.60–97.90)	42.35 (25.40–72.75)	**<0.001**
FL	67.20 (60.97–71.06)	67.51 (61.50–69.90)	0.625
FL percentile	41.60 (22.10–75.00)	38.90 (25.15–54.85)	0.329
EFW	2616.00 (1943.00–3054.00)	2409.00 (1905.50–2791.00)	0.059
EFW percentile	69.50 (37.40–91.20)	43.05 (26.05–64.90)	**<0.001**
MCA DT	0.31 (0.29–0.33)	0.30 (0.27–0.32)	0.072
MCA DN	34.06 (27.70–41.36)	36.59 (30.73–43.62)	**0.048**
MCA D	8.41 (6.02–11.10)	8.69 (5.32–12.47)	0.850
MCA DDA	6.67 (5.02–8.20)	7.05 (5.21–8.39)	0.444
UA S/D	2.43 (2.14–2.92)	2.54 (2.17–2.92)	0.473
UA PI	0.88 (0.74–1.05)	0.92 (0.79–1.05)	0.222
MCA PSV	48.69 (40.60–58.28)	48.96 (42.46–59.29)	0.439
PI MCA	1.77 (1.56–2.07)	1.79 (1.43–2.31)	0.600
S/D MCA	5.44 (4.29–7.30)	5.25 (4.13–9.74)	0.835
CPR	2.11 (1.70–2.48)	2.19 (1.54–2.54)	0.990
UCR	0.47 (0.40–0.59)	0.46 (0.39–0.65)	0.990
DV PI	0.49 (0.37–0.65)	0.54 (0.39–0.70)	0.159
DV TAMXV	42.13 (34.05–59.84)	39.30 (27.80–48.60)	**0.030**

* The Mann–Whitney U test was performed. BPD: biparietal diameter, HC: head circumference, AC: abdominal circumference, FL: femur length, EFW: estimated fetal weight, UA: umbilical artery, S/D: systolic/diastolic, PI: pulsatility index, MCA: middle cerebral artery, DDA: diastolic deceleration area, PSV: peak systolic velocity, DV: ductus venosus, CPR: cerebroplacental ratio, UCR: umbilicocerebral ratio, TAMXV: time-averaged maximum velocity.

**Table 3:** Delivery mode and neonatal outcomes in diabetic and control groups.

	GDM (*n* = 83)		Control (*n* = 92)		*p* *
	n	%	n	%	
Cesarean section	66	79.5	60	65.2	**0.035**
Neonatal respiratory morbidity	4	4.8	9	9.8	0.211
Hypoglycemia	1	1.2	1	1.1	0.942
Hypocalcemia	2	2.4	0	0.0	0.224
Hyperbilirubinemia (Need for phototherapy)	1	1.2	3	3.3	0.623
Need for CPAP	1	1.2	2	2.2	0.622
Need for mechanical ventilation	1	1.2	2	2.2	0.622
CANO	7	8.4	10	10.9	0.587
NICU admission	7	10.1	11	15.1	0.378
Asphyxia	1	1.2	1	1.1	0.942
Neonatal death	1	1.2	0	0.0	0.474
5th minute Apgar score <7	0	0	0	0	NA

* Chi-square test was used. CPAP: continuous positive airway pressure, NICU: neonatal intensive care unit, CANO: Composite adverse neonatal outcomes; NA: Not Applicable.

**Table 4:** Correlations between MCA DDA and other clinical and Doppler parameters in the patient group.

	MCA DDA	
	r *	*p*
BPD	**0.246**	**0.025**
BPD percentile	0.001	0.998
HC	0.210	0.056
HC percentile	−0.090	0.421
AC	0.196	0.076
AC percentile	−0.158	0.153
FL	**0.294**	**0.007**
FL percentile	0.025	0.820
EFW	**0.236**	**0.032**
EFW percentile	−0.101	0.361
UA S/D	−0.040	0.722
UA PI	−0.002	0.986
MCA PSV	**0.570**	**<0.001**
MCA PI	**−0.274**	**0.013**
MCA S/D	−0.205	0.063
CPR	−0.191	0.085
UCR	0.191	0.085
DV PI	−0.054	0.630
GA at delivery	0.051	0.648
Birth weight	**−0.260**	**0.018**
APGAR Score at 1 th minute	−0.106	0.342
APGAR Score at 5th minute	−0.168	0.128
NICU stay duration	**0.880**	**0.021**

* Spearman correlation analysis was applied. BPD: biparietal diameter, HC: head circumference, AC: abdominal circumference, FL: femur length, EFW: estimated fetal weight, UA: umbilical artery, S/D: systolic/diastolic, PI: pulsatility index, MCA: middle cerebral artery, DDA: diastolic deceleration area, PSV: peak systolic velocity, DV: ductus venosus, CPR: cerebroplacental ratio, UCR: umbilicocerebral ratio, GA: Gestational age, NICU: neonatal intensive care unit.

**Table 5:** Correlation of neonatal outcomes with Doppler parameters in the patient group.

		GA at Delivery	Birth Weight	APGAR Score at 1st Minute	APGAR Score at 5th Minute
MCA DT	r	0.151	**−0.237**	−0.050	−0.103
	*p*	0.172	**0.031**	0.654	0.353
MCA DN	r	0.028	−0.188	−0.162	**−0.248**
	*p*	0.802	0.089	0.143	**0.024**
MCA D	r	−0.031	−0.210	−0.107	−0.177
	*p*	0.780	0.057	0.334	0.109
MCA DDA	r	0.051	**−0.260**	−0.106	−0.168
	*p*	0.648	**0.018**	0.342	0.128
UA S/D	r	−0.015	−0.152	0.002	−0.128
	*p*	0.890	0.169	0.986	0.247
UA PI	r	−0.025	−0.151	0.124	−0.020
	*p*	0.819	0.174	0.264	0.858
MCA PI	r	−0.079	**0.235**	−0.017	−0.075
	*p*	0.482	**0.034**	0.876	0.505
MCA S/D	r	−0.060	**0.262**	−0.007	−0.053
	*p*	0.591	**0.017**	0.950	0.632
CPR	r	−0.067	**0.244**	−0.140	−0.071
	*p*	0.548	**0.027**	0.209	0.526
UCR	r	0.067	**−0.244**	0.140	0.071
	*p*	0.548	**0.027**	0.209	0.526
DV PI	r	**−0.239**	−0.039	−0.082	−0.203
	*p*	**0.030**	0.724	0.461	0.066

Spearman correlation analysis was applied. UA: umbilical artery, S/D: systolic/diastolic, PI: pulsatility index, MCA: middle cerebral artery, DDA: diastolic deceleration area, PSV: peak systolic velocity, DV: ductus venosus, CPR: cerebroplacental ratio, UCR: umbilicocerebral ratio.

**Table 6:** Multivariable linear regression analysis of factors associated with MCA DDA.

Variable	B	Std. Error	Beta	t	*p*
Constant	−7.775	4.779	-	−1.627	0.106
GDM group	0.658	0.906	0.072	0.725	0.469
Maternal age	−0.035	0.068	−0.040	−0.524	0.601
BMI	−0.107	0.067	−0.124	−1.611	0.109
Insulin use	0.569	0.984	0.056	0.578	0.564
Gestational age	**0.561**	**0.118**	**0.343**	**4.769**	**<0.001**

R^2^ = 0.132; Adjusted R^2^ = 0.107; F(5,169) = 5.151; *p* < 0.001. BMI: body mass index, CI: confidence interval, GDM: gestational diabetes mellitus, MCA DDA: middle cerebral artery diastolic deceleration area.

**Table 7:** Multiple logistic regression analysis for CANO.

Variable	B	Std. Error	Wald	*p*	OR (Exp(B))	95% CI Lower	95% G CI Upper
MCA DDA	−0.048	0.120	0.162	0.687	0.953	0.753	1.205
Maternal age	0.006	0.057	0.011	0.917	1.006	0.899	1.126
BMI	0.024	0.056	0.185	0.667	1.024	0.919	1.142
Gestational age	0.089	0.115	0.591	0.442	1.093	0.872	1.370
GDM group	0.554	0.834	0.440	0.507	1.739	0.339	8.920
Insulin use	0.096	0.953	0.010	0.920	1.101	0.170	7.132

BMI: body mass index, CI: confidence interval, GDM: gestational diabetes mellitus, MCA DDA: middle cerebral artery diastolic deceleration area, OR: odds ratio, CANO: composite adverse neonatal outcome.

## Data Availability

Data are not publicly available due to ethical restrictions. Further inquiries can be directed to the corresponding author.

## References

[1] Crowther C.A., Hiller J.E., Moss J.R., McPhee A.J., Jeffries W.S., Robinson J.S. (2005). Effect of Treatment of Gestational Diabetes Mellitus on Pregnancy Outcomes. N. Engl. J. Med..

[2] Acar T.T., Asker L., Guven H.K., Kurt G.Y., Ulusoy C.O., Yilmaz E., Yilmaz Z.V. (2025). Fetal Hepatic Artery Doppler in Pregnant Women With Gestational Diabetes Mellitus. J. Clin. Ultrasound.

[3] Lund A., Ebbing C., Rasmussen S., Kiserud T., Kessler J. (2018). Maternal diabetes alters the development of ductus venosus shunting in the fetus. Acta Obstet. Gynecol. Scand..

[4] Leung W.C., Lam H., Lee C.P., Lao T.T. (2004). Doppler study of the umbilical and fetal middle cerebral arteries in women with gestational diabetes mellitus. Ultrasound Obstet. Gynecol..

[5] Graupner O., Rath C., Lecker L., Ritgen J., Haller B., Enzensberger C. (2025). Fetomaternal Doppler sonography for the prediction of perinatal outcome in term pregnancies complicated by gestational diabetes mellitus: Does it have potential?. Ultrasound Int. Open.

[6] Cardinali F., Panunzi C., D’ANtonio F., Khalil A., Spinillo A., Arossa A., Familiari A., Pagani G., Resta S., Rizzo G. (2024). Role of Cerebroplacental Ratio in Predicting the Outcome of Pregnancies Complicated by Diabetes. Fetal Diagn. Ther..

[7] Patey O., Carvalho J.S., Thilaganathan B. (2019). Perinatal changes in fetal cardiac geometry and function in diabetic pregnancy at term. Ultrasound Obstet. Gynecol..

[8] Ezveci H., Doğru Ş., Yaman F.K. (2025). Predictability of fetal pulmonary artery Doppler on neonatal outcomes in pregnant women with gestational diabetes mellitus. Congenit. Anom..

[9] Omeroglu I., Golbasi H., Bayraktar B., Golbasi C., Karaca S.Y., Demircan T., Ekin A. (2023). Predicting adverse perinatal outcomes with fetal modified myocardial performance index and epicardial fat tissue thickness in diabetes-complicated pregnancies. Eur. Rev. Med. Pharmacol. Sci..

[10] Ignatov P.N., Neykova K.K., Yordanova-Ignatova T. (2023). Diastolic deceleration area in the fetal MCA: A new Doppler parameter. J. Matern. Fetal Neonatal. Med..

[11] Metzger B.E., Gabbe S.G., Persson B., Buchanan T.A., Catalano P.A., Damm P., Dyer A.R., de Leiva A., Hod M., International Association of Diabetes and Pregnancy Study Groups Consensus Panel (2010). International association of diabetes and pregnancy study groups recommendations on the diagnosis and classification of hyperglycemia in pregnancy. Diabetes Care.

[12] Serbetci H., Tanacan A., Zorlu U., Karatas E., Okutucu G., Soganci E., Kara O., Sahin D. (2025). Expanding Doppler Velocimetry Horizons: Predicting Hypoxia and Adverse Perinatal Outcomes Using Fetal Middle Cerebral Artery Diastolic Deceleration Area. J. Clin. Ultrasound.

[13] Khalil A., Sotiriadis A., D’Antonio F., Costa F.D.S., Odibo A., Prefumo F., Papageorghiou A.T., Salomon L.J. (2024). ISUOG Practice Guidelines: Performance of third-trimester obstetric ultrasound scan. Ultrasound Obstet. Gynecol..

[14] Hadlock F.P., Harrist R.B., Sharman R.S., Deter R.L., Park S.K. (1985). Estimation of fetal weight with the use of head, body, and femur measurements-A prospective study. Am. J. Obstet. Gynecol..

[15] Ganor Paz Y., Barzilay E., Saied Idriss S., Murray-Davis B., Melamed N., Ray J., Geary M., McDonald S., Barrett J., Mawjee K. (2022). Association of the Cerebro-Placental Ratio With Adverse Outcomes in Pregnancies Affected by Gestational Diabetes Mellitus. J. Ultrasound Med..

[16] Dantas A.M.A., Palmieri A.B.S., Vieira M.R., Souza M.L.R., Silva J.C. (2019). Doppler ultrasonographic assessment of fetal middle cerebral artery peak systolic velocity in gestational diabetes mellitus. Int. J. Gynecol. Obstet..

[17] Shabani Zanjani M., Nasirzadeh R., Fereshtehnejad S.M., Yoonesi Asl L., Alemzadeh S.A.P., Askari S. (2014). Fetal cerebral hemodynamic in gestational diabetic versus normal pregnancies: A Doppler velocimetry of middle cerebral and umbilical arteries. Acta Neurol. Belg..

[18] Cetin C., Takmaz T., Dolanbay M., Kutuk M.S. (2023). The Value of Fetal Cerebro-umbilical Doppler Indices in Predicting Umbilical Blood Gas Abnormalities and Apgar Score in Diabetic Pregnant Women. Haseki Tip Bul..

[19] Stuart A., Amer-wåhlin I., Gudmundsson S., Maršál K., Thuring A., Källen K. (2010). Ductus venosus blood flow velocity waveform in diabetic pregnancies. Ultrasound Obstet. Gynecol..

[20] Lu J.L., Capponi A., Mappa I., Maneschi F., Rizzo G. (2021). Effects of gestational diabetes mellitus on ductus venosus shunting during the third trimester. Prenat. Cardiol..

[21] Karabay G. (2025). Relationship Between Adverse Neonatal Outcomes and Diastolic Deceleration Area on Fetal MCA Doppler in Patients with Late Fetal Growth Restriction. SiSli Etfal Hastan. Tip Bul./Med. Bull. Sisli Hosp..

[22] Mlodawski J., Mlodawska M., Plusajska J., Detka K., Michalska A., Swiercz G., Sikorski M. (2021). Repeatability and reproducibility of quantitative cervical strain elastography (E-Cervix) in pregnancy. Sci. Rep..

[23] Gamrah M.A., Xu J., El Sawy A., Aguib H., Yacoub M., Parker K.H. (2020). Mechanics of the dicrotic notch: An acceleration hypothesis. Proc. Inst. Mech. Eng. Part H J. Eng. Med..

[24] Politi M.T., Ghigo A., Fernández J.M., Khelifa I., Gaudric J., Fullana J.M., Lagrée P.-Y. (2016). The dicrotic notch analyzed by a numerical model. Comput. Biol. Med..

